# Food labelling

**DOI:** 10.1093/eurpub/ckac129.002

**Published:** 2022-10-25

**Authors:** N Gokani

**Affiliations:** School of Law, University of Essex, Colchester, UK

## Abstract

The Commission has undertaken to regulate the information provided to consumers on food. In particular, it intends to propose mandatory front-of-pack nutrition labelling (FoPNL) for food by the end of 2022. Even though nutrition labelling has been regulated by the EU since 1990, the tactics of industry, with the support of a minority of Member States, have prevented, so far, the adoption of a single, EU-wide, mandatory FoPNL scheme. Questions therefore arise regarding the extent to which the EU may secure agreement for the adoption of an EU-wide scheme which could effectively contribute to a high level of public health protection.

